# Paradigm shift: new concepts for HCN4 function in cardiac pacemaking

**DOI:** 10.1007/s00424-022-02698-4

**Published:** 2022-05-13

**Authors:** Konstantin Hennis, Martin Biel, Stefanie Fenske, Christian Wahl-Schott

**Affiliations:** 1 Center for Drug Research, Department of Pharmacy, Ludwig-Maximilians-Universität München, 81377 Munich, Germany; 2 German Center for Cardiovascular Research (DZHK), Partner Site Munich Heart Alliance, 80802 Munich, Germany; 3 Institute for Neurophysiology, Hannover Medical School, 30625 Hannover, Germany

**Keywords:** Sinoatrial node (SAN), Chronotropic effect, Heart rate regulation, Autonomic nervous system, Hyperpolarization-activated cyclic nucleotide–gated channels, HCN4 channel

## Abstract

Hyperpolarization-activated cyclic nucleotide–gated (HCN) channels are the molecular correlate of the I_f_ current and are critically involved in controlling neuronal excitability and the autonomous rhythm of the heart. The HCN4 isoform is the main HCN channel subtype expressed in the sinoatrial node (SAN), a tissue composed of specialized pacemaker cells responsible for generating the intrinsic heartbeat. More than 40 years ago, the I_f_ current was first discovered in rabbit SAN tissue. Along with this discovery, a theory was proposed that cyclic adenosine monophosphate–dependent modulation of I_f_ mediates heart rate regulation by the autonomic nervous system—a process called chronotropic effect. However, up to the present day, this classical theory could not be reliably validated. Recently, new concepts emerged confirming that HCN4 channels indeed play an important role in heart rate regulation. However, the cellular mechanism by which HCN4 controls heart rate turned out to be completely different than originally postulated. Here, we review the latest findings regarding the physiological role of HCN4 in the SAN. We describe a newly discovered mechanism underlying heart rate regulation by HCN4 at the tissue and single cell levels, and we discuss these observations in the context of results from previously studied HCN4 mouse models.

## Introduction

Hyperpolarization-activated cyclic nucleotide–gated (HCN) channels are transmembrane proteins that are expressed mainly in the cardiovascular system and central nervous system [7]. They represent a specific class of ion channels within the superfamily of pore-loop cation channels. HCN channels are the molecular correlate of the mixed Na^+^/K^+^ current I_f_, which is also termed I_h_/I_q_ or simply referred to as “pacemaker current.” The latter term reflects the important roles of HCN channels in controlling neuronal excitability and the autonomous rhythm of the heart. The most remarkable characteristic of HCN channels lies in activation by membrane hyperpolarization, which is in stark contrast to the depolarization-dependent activation of other voltage-gated ion channels. As a consequence, HCN channels are open and mediate a depolarizing inward current at membrane potentials negative to the channel’s reversal potential, which is about −40 mV under physiological ion concentrations [7]. This voltage range corresponds, for instance, to the diastolic membrane potentials in pacemaker cells of the cardiac conduction system (CCS). Another key feature is the modulation of HCN channel activity by cyclic nucleotides such as cyclic adenosine monophosphate (cAMP). It has been shown that the intracellular cyclic nucleotide–binding domain (CNBD) [78] exerts an inhibiting effect on the transmembrane core of the channel. Binding of cyclic nucleotides to the CNBD leads to a conformational change that is propagated through the C-linker and ultimately relieves inhibition of the transmembrane region, thereby facilitating channel opening [14, 60, 64, 74]. In this way, changes in the intracellular cAMP concentration significantly regulate HCN channel activity within the physiological range of membrane potentials [16].

The HCN channel subfamily comprises four members termed HCN1–HCN4 [6, 7, 44, 47]. Profound differences between the four isoforms are observed in their biophysical properties including cAMP modulation and gating kinetics. HCN2 and HCN4 channels are highly sensitive to cAMP, while HCN1 activity is only weakly influenced, and HCN3, although containing an intact CNBD, is not affected by cAMP [6, 26]. Regarding the activation kinetics, HCN1 can be classified as the fastest channel followed by HCN2 and HCN3. HCN4 displays by far the slowest gating kinetics among the four subtypes [6].

**Fig. 1:**
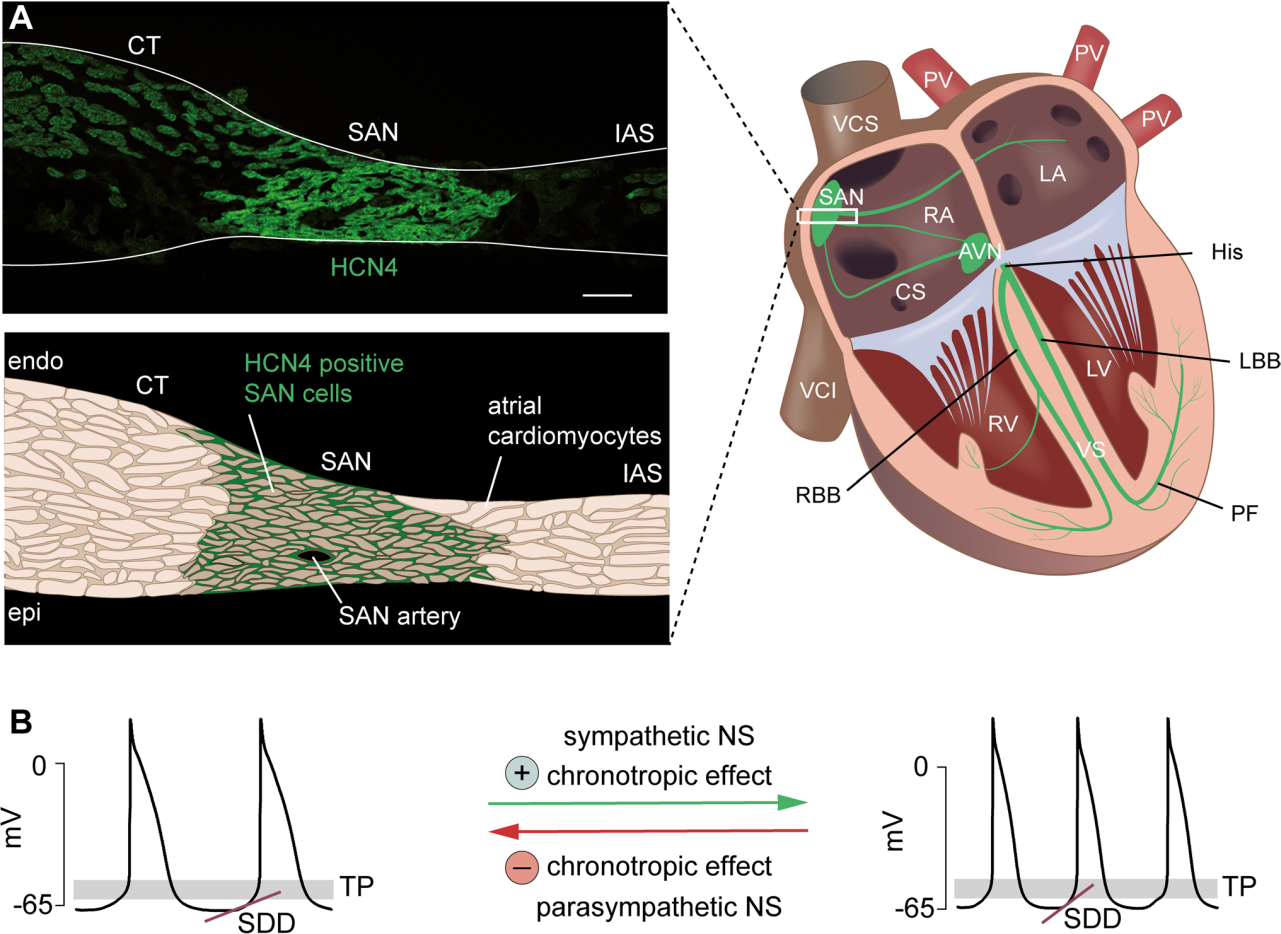
HCN4 is expressed in the sinoatrial node. (A) Right, schematic diagram of the cardiac conduction system (green). The primary pacemaker site is the sinoatrial node (SAN). The atrioventricular node (AVN) is the only electrically conductive connection between the atria and the ventricles. The bundle of His (His) splits into a left and right bundle branch (LBB/RBB, left/right bundle branch), which spread out to the left and right Purkinje fiber (PF) network. Abbreviations: LA, left atrium; RA, right atrium; PV, pulmonary veins; VCS, superior vena cava; VCI, inferior vena cava; CS, coronary sinus; RV, right ventricle; LV, left ventricle; VS, ventricular septum. (A) Left, upper panel: distribution of HCN4 (green) in a transverse section of the murine SAN. HCN4 is expressed across the entire SAN region. Abbreviations: CT, crista terminalis; IAS, interatrial septum. Scale bar: 100 µm. Lower panel: schematic illustration of the original image shown in the upper panel. (B) Action potential recordings of isolated SAN cells demonstrating the chronotropic effect at the single cell level. Input from the sympathetic nervous system accelerates SDD and increases the firing rate of pacemaker cells, whereas input from the parasympathetic nervous system slows down SDD and decelerates the firing rate. Abbreviations: SDD, slow diastolic depolarization; TP, threshold potential; NS, nervous system

The I_f_ current was first discovered in 1979 [9] in rabbit sinoatrial node (SAN) tissue. Along with this discovery, a theory was proposed that modulation of I_f_ mediates heart rate (HR) regulation by the autonomic nervous system (ANS). This classical hypothesis assumes that the chronotropic effect, i.e., the acceleration or deceleration of HR following sympathetic or vagal activity, respectively, is mediated by an increase or decrease in I_f_ as a response to rising or falling intracellular cAMP levels [9, 15–17]. Despite being under continuous scientific dispute, this theory became prominent and finally reached the textbooks of physiology and pharmacology. However, after the discovery of the I_f_ current, it took almost 20 further years until the four members of the HCN channel family were cloned [44]. This achievement finally provided the basis for studying the channels at the molecular level and for creating genetically modified animal models with lacking or mutated HCN channels. In all mammalian species studied so far, HCN4 was found to be the main HCN channel isoform in the SAN (Fig. 1A). Therefore, many groups became interested in specifically investigating the physiological role of the HCN4 subtype using genetically engineered mouse models. Interestingly, these studies led to partially contradictory conclusions about the role of HCN4 and its cAMP-dependent regulation (CDR) in SAN function, and up to the present day, the abovementioned classical theory could not be directly confirmed in vivo. Just recently, new concepts emerged confirming that HCN4 channels play an important role in HR regulation. However, these concepts provide evidence for a completely different cellular mechanism than originally postulated. In this review, we highlight the latest findings regarding the physiological role of CDR of HCN4 in HR regulation, and we discuss these observations in the context of results from previously studied HCN4 mouse models.

## Cardiac conduction system and putative mechanism for the chronotropic effect

As mentioned above, HCN4 is expressed in the heart and is particularly important in the SAN, which is the primary pacemaker to control heart rate (HR) under physiological conditions [7, 8, 26, 34, 42, 47]. The main characteristic feature of SAN pacemaker cells that enables spontaneous firing of action potentials is the slow diastolic depolarization (SDD; Fig. 1B). This phase of the pacemaker cycle is initiated after action potential termination, when the membrane potential is most negative (maximum diastolic potential, MDP). During SDD, the pacemaker cells do not remain at a stable resting membrane potential (RMP), but instead slowly depolarize the membrane until a threshold potential (TP) is reached at which the upstroke of the next action potential is initiated. The rate of membrane depolarization during SDD critically controls the firing frequency of pacemaker cells and consequently determines the heart rate [27, 47].

For the ability to spontaneously generate rhythmic action potentials, a strictly timed interplay of different ionic membrane currents is required. During the initial phase of SDD, the depolarizing I_f_ current drives the membrane potential toward the threshold for opening of voltage-gated T-type Ca_V_3.1 and L-type Ca_V_1.3 channels. The resulting combination of inward currents produces a further depolarization during late SDD, leading to an additional opening of L-type Ca_V_1.2 channels. Subsequently, I_Ca,L_ is mainly responsible for generating the action potential upstroke and overshoot. The positive membrane potential then leads to inactivation of voltage-gated calcium currents and activation of delayed rectifier potassium currents. The outward K^+^ currents I_K,r_ and I_K,s_ finally cause membrane repolarization and return to the MDP [47, 53].

To enable adaption to varying physical activity, HR is regulated by the autonomic nervous system (ANS). The ANS comprises the sympathetic nervous system (SNS) and the parasympathetic nervous system (PSNS). Both branches of the ANS innervate the SAN [58] and regulate the firing rate of pacemaker cells by altering the slope of SDD−a process called chronotropic effect (Fig. 1B). Upon activation of the SNS, norepinephrine is released from sympathetic nerve terminals and binds to G_s_ protein-coupled beta-1-adrenergic receptors located on the surface of SAN pacemaker cells. Subsequently, adenylyl cyclases inside the cell are activated, which leads to an increase in the cytosolic concentration of the second messenger cAMP [5]. Reversely, upon PSNS activation, acetylcholine is released from vagal nerve terminals and activates G_i_ protein-coupled M2-muscarinic acetylcholine receptors. This causes inhibition of adenylyl cyclases, resulting in decreased intracellular cAMP levels. The changes in the cAMP concentration eventually affect the rate of SDD and thereby accelerate or decelerate the heart rate, which is referred to as positive or negative chronotropic effect, respectively.

HCN4 is present throughout the entire SAN region (Fig. 1A), where it mediates approximately 75% of the I_f_ current [29, 30, 54]. As outlined above, HCN4 channel activity is substantially controlled by intracellular cAMP concentrations and is therefore directly regulated by the ANS. Moreover, the channels are open throughout the pacemaker cycle [59] and conduct a depolarizing inward current at hyperpolarized membrane potentials spanning the range of SDD. Theoretically, these properties would be ideal to significantly alter the rate of SDD in response to changes in the intracellular cAMP concentration. However, many of the proteins involved in the pacemaker process are known to be direct or indirect cAMP targets, including L-type Ca^2+^ channels and delayed rectifier potassium channels. Therefore, there are various potential candidates to mediate the chronotropic effect at the subcellular level, and the main contributor to HR regulation in SAN pacemaker cells has not yet been clearly identified [27]. In particular, a possible role of HCN4 CDR in mediating the chronotropic effect is highly controversial and is, therefore, still a subject of current research.

## HCN4 mouse models

Over the past 20 years, many different HCN4 mouse models (Table 1) have been created to investigate the role of this particular ion channel in SAN function [11, 31]. In 2003, the first HCN4 knockout mouse model was created [66]. Interestingly, this study revealed that global HCN4–deficiency causes embryonic lethality, which is attributable to a severely diminished I_f_ current during cardiac development. The authors reported that in embryonic HCN4^−/−^ hearts, HR was strongly reduced by approximately 40%. Furthermore, HR as well as action potential firing rate in isolated embryonic HCN4^−/−^ cardiomyocytes could not be increased by cAMP. Similar results were obtained in a different study investigating a mouse model with only a single amino acid exchange (R669Q) in the HCN4 CNBD [25]. This mutation abolishes CDR of HCN4, while leaving all other functional properties unaffected. Most importantly, also, HCN4R669Q mice are embryonically lethal, which strongly suggests that basal cAMP-dependent activation of HCN4 is a general prerequisite for the physiological function of the channel and that preventing CDR alone has similar functional effects as complete knockout of HCN4. The main findings from this study include that embryonic HCN4R669Q hearts displayed significantly reduced HRs and abolished responsiveness to catecholaminergic stimulation. Together, these studies supported the classical theory that CDR of HCN4 is responsible for mediating the chronotropic effect. However, the conclusions that can be drawn from these results are limited, since in embryonic hearts, the SAN and CCS are not yet completely developed.

**Table 1:** Action potential characteristics and cardiac phenotype of HCN4 mouse models

Reference	Firing properties (single cells)	Membrane potential (MDP/RMP)	Firing frequency (single cells)	Heart rate (HR)	Cardiac phenotype
HCN4 knockout					
Stieber et al. [66] HCN4 knockout Global	Not reported	Not reported	Not reported	Not reported	Embryonic lethality
Stieber et al. [66] HCN4 knockout Heart-specific	Isolated embryonic cardiomyocytes: Immature pacemaker potentials	Unchanged KO: − 65 mV WT: − 70 mV	Isolated embryonic cardiomyocytes: Firing rate normal	Isolated embryonic hearts: HR reduced	Embryonic lethality Isolated embryonic hearts: Chronotropic effect absent
Herrmann et al. [29] HCN4 knockout Global Tamoxifen-inducible	Isolated SAN cells: Large fraction of quiescent cells (90%)	Hyperpolarized Δ = − 8 mV	Spontaneously active cells: Firing rate normal	Adult mice: HR unchanged	Sinus pauses Chronotropic effect preserved
Hoesl et al. [33] HCN4 knockout Conduction system-specific	Isolated SAN cells: large fraction of quiescent cells	Hyperpolarized	Spontaneously active cells: Firing rate normal	Adult mice: HR unchanged	Sinus pauses Chronotropic effect preserved
Baruscotti et al. [3] HCN4 knockout Heart-specific Tamoxifen-inducible	Isolated SAN cells: Successive reduction in firing frequency after induction of KO	Not reported	Isolated SAN cells: Firing rate reduced	Adult mice: HR reduced	Bradycardia, AV block, sinus arrest Chronotropic effect preserved
Mesirca et al. [52] hHCN4-AYA (transgenic, dominant-negative) Heart-specific Inducible (doxycycline-withdrawal)	Isolated SAN cells: Delayed after depolarizations (DADs)	Unchanged Mutant: − 64 mV WT: − 60 mV	Isolated SAN cells: Firing rate reduced	Adult mice: HR reduced	Bradycardia, AV block, ventricular tachycardia Chronotropic effect preserved
HCN4 overexpression or knockdown					
Kozasa et al. [37] HCN4 overexpression Global (Heterozygous, without doxycycline)	Isolated SAN cells: Regular firing pattern	Not reported	Isolated SAN cells: Firing rate normal	Adult mice (HZ): HR unchanged	Adult mice (HZ): HRV reduced
Kozasa et al. [37] HCN4 knockdown Global (Heterozygous, doxycycline-inducible)	Isolated SAN cells: Irregular firing pattern (quiescent cells excluded)	Not reported	Isolated SAN cells: Firing rate reduced	Adult mice (HZ): HR reduced	Adult mice (HZ): Bradycardia, sinus arrhythmia, HRV increased Chronotropic effect preserved
cAMP-insensitive HCN4 channels					
Harzheim et al. [25] HCN4R669Q, knock-in, cAMP-insensitive Global	Isolated embryonic cardiomyocytes: Slow but regular firing pattern	Hyperpolarized Mutant: − 83 mV WT: − 74 mV	Isolated embryonic cardiomyocytes: Firing rate reduced	Isolated embryonic hearts: HR reduced Adult mice (HZ): HR unchanged	Embryonic lethality Chronotropic effect absent
Alig et al. [1] hHCN4-573X, transgenic, cAMP-insensitive Heart-specific Inducible (dox-withdrawal)	Isolated SAN cells: Heterogeneous firing patterns (quiescent, intermittent beating, arrhythmic)	Depolarized Mutant: − 51 mV WT: − 58 mV (quiescent cells excluded)	Spontaneously active SAN cells: Firing rate reduced	Adult mice: HR reduced	Bradycardia Chronotropic effect preserved
Fenske et al. [22] HCN4FEA, knock-in, cAMP-insensitive Global	Isolated SAN cells: Periodic changes between firing and nonfiring	Unchanged during firing Hyperpolarized during nonfiring	Isolated SAN cells: Normal rate during firing	Adult mice: HR reduced	Bradycardia, sinus dysrhythmia, escape arrhythmias Chronotropic effect preserved

To allow for investigation of adult animals lacking HCN4, several inducible, global [29], or cardiac-specific [3, 33] HCN4 knockout mouse models were created. In contrast to the findings from embryonic hearts, the positive chronotropic effect, as determined by responsiveness to beta-adrenergic stimulation, was fully preserved in all these mouse models. A further study, in which a transgenic approach was used to achieve cardiac-specific silencing of I_f_ (hHCN4–AYA mice), confirmed these results [52]. In addition, the majority of the studies reported severe bradycardia and sinus dysrhythmia, suggesting that the absence of HCN4 gives rise to intrinsically reduced HRs and overshooting parasympathetic responses [3, 52]. To further study the contribution of HCN4 to autonomic HR regulation, Kozasa et al. created a transgenic mouse model with inducible, global overexpression or knockdown of HCN4 [37]. Interestingly, HCN4 overexpression did not induce tachycardia but reduced heart rate variability and attenuated HR-lowering responses to cervical vagus nerve stimulation in vivo. In contrast, conditional knockdown of HCN4 led to pronounced bradycardia and overshooting responses to vagus nerve stimulation. HR responses to beta-adrenergic stimulation, however, were not altered in both groups of mice. To investigate the role of HCN4 CDR more directly, a further transgenic mouse model was created [1]. These mice are characterized by cardiac-specific expression of a mutant construct (hHCN4-573X) based on a mutation originally identified in a human patient with idiopathic sinus node dysfunction [65]. The mutation leads to a large C-terminal truncation that, besides other regulatory domains, includes the CNBD of HCN4 and thereby suppresses cAMP-sensitivity of the channel in a dominant-negative manner. Also, in these mice, the authors reported a significant reduction in HR at rest and during exercise, whereas the relative range of HR regulation was preserved.

To study the role of HCN4 CDR even more precisely, the cAMP-insensitive HCN4FEA knock-in mouse model was generated [22]. HCN4FEA animals carry three point mutations in the *Hcn4* gene, resulting in global expression of mutant HCN4 channels that contain two amino acid exchanges in the CNBD (R669E, T670A) and one in the C-linker (Y527F). The R669E and T670A mutations completely disrupt binding of cAMP to the CNBD, resulting in the loss of CDR of the channel. However, as mentioned above, it has been demonstrated that cAMP-dependent activation of HCN4 is fundamentally required for the physiological function of the channel and that mice with globally abolished HCN4 CDR die during embryonic development [25]. This is possibly caused by the fact that in the complete absence of cAMP, or in mutant channels that cannot be activated by cAMP, the activation thresholds of the channel are more negative than the MDP of pacemaker cells, which essentially causes a functional knockout of HCN4 [22, 29]. To avoid such experimental limitations, the Y527F mutation in the C-linker was additionally introduced. This mutation simulates channel activation by basal cAMP levels, reflected by a constant shift of the activation curve towards more positive potentials and moderate acceleration of gating kinetics, altogether preventing embryonic lethality. These characteristics make the HCN4FEA mouse an ideal animal model to selectively study the physiological significance of HCN4 CDR for the cardiac pacemaker process and HR regulation by the ANS [22].

## Nonfiring pacemaker cells in the SAN network

**Fig. 2:**
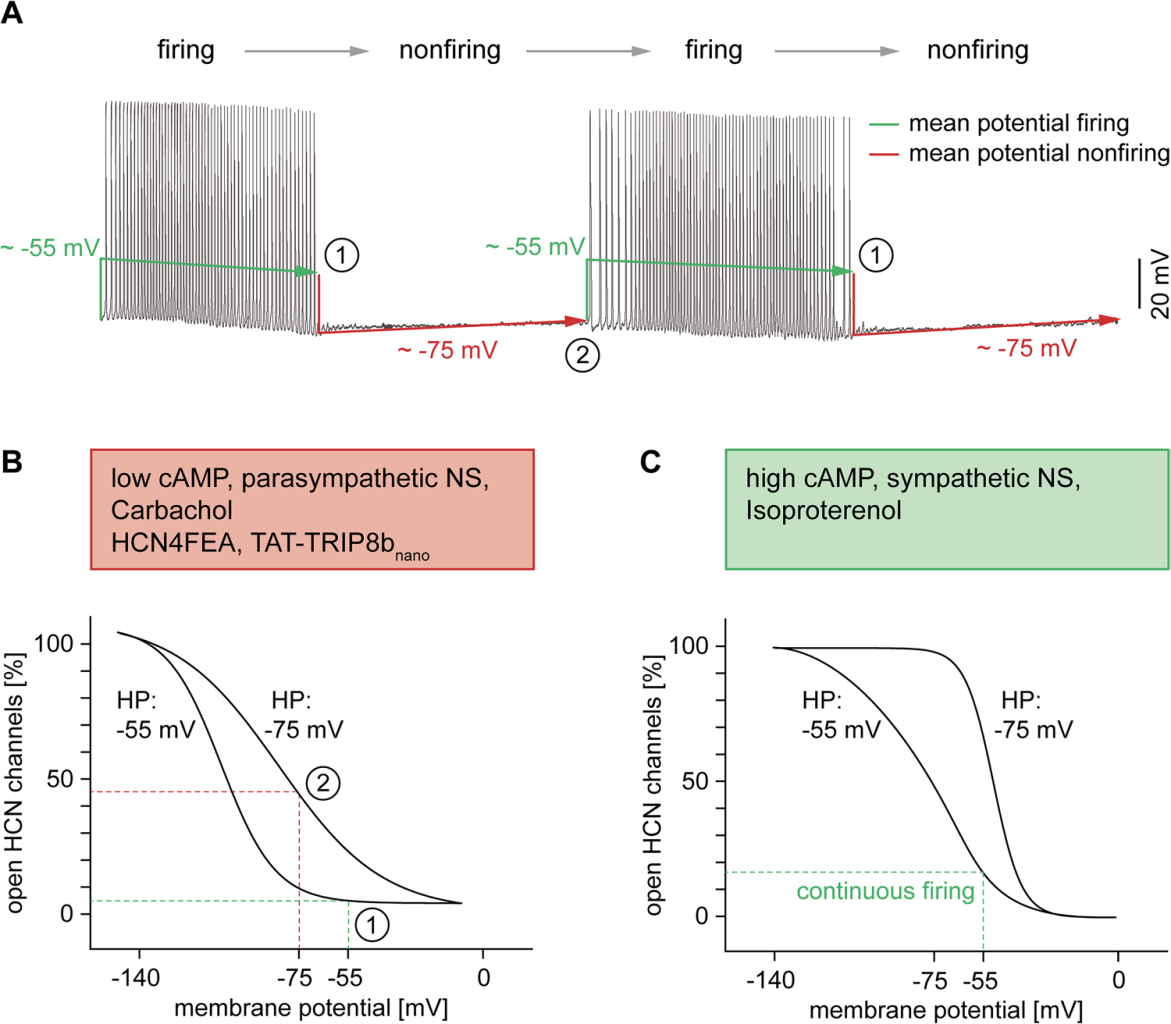
CDR and hysteresis of HCN4 control the firing mode of SAN cells. (**A**) Action potential recording of an isolated pacemaker cell showing the typical alternation between firing and nonfiring. The mean membrane potential is more depolarized during firing (~ − 55 mV, green line) and more hyperpolarized during nonfiring (~ − 75 mV, red line). At the same time, slow drifts in membrane potential occur. The firing mode is characterized by a slow, progressive hyperpolarization (Δ =  − 7 mV) until firing stops (1), leading to an abrupt drop to significantly more hyperpolarized potentials. Conversely, during nonfiring, a slow and progressive depolarization occurs until the threshold for firing is reached (2), and the membrane potential abruptly jumps to substantially more depolarized values. Due to hysteresis of HCN4, the changes in membrane potential have important consequences for the voltage-dependent activation of the channel. (**B**) Original steady-state activation curves recorded from HCN4 channels heterologously expressed in HEK239 cells without cAMP in the intracellular solution. The long-lasting, mean membrane potentials during firing (− 55 mV) and nonfiring (− 75 mV) are mimicked by the holding potential (HP). At a relatively depolarized holding potential of − 55 mV, the activation curve is positioned at extremely hyperpolarized voltages (left curve). Conversely, at a relatively hyperpolarized holding potential of − 75 mV, the activation curve is positioned at extremely depolarized voltages (right curve). Points (1) and (2) on the activation curves reflect the time points (1) and (2) of the action potential measurements shown in panel (A). At the end of firing (1), the activation curve is shifted to the left and the membrane potential is relatively positive, resulting in a small number of open HCN4 channels. The consequent lack of a sufficiently depolarizing I_f_ current causes or supports the transition of pacemaker cells to the nonfiring mode. At the end of nonfiring (2), the activation curve is shifted to the right and the membrane potential is relatively negative, leading to a substantial increase in the number of open HCN4 channels, thereby causing or supporting the return of pacemaker cells to the firing mode. (**C**) Original activation curves of HCN4 channels recorded in the presence of 100 µM cAMP in the intracellular solution. Compared to panel (**B**), both curves are markedly shifted to the right. Under comparable conditions, SAN cells do not switch into the nonfiring mode, and the mean membrane potential permanently remains at depolarized values (− 55 mV), leading to a sufficient number of open HCN4 channels to maintain continuous firing

The main finding from this study was the discovery of a novel and thus far uncharacterized nonfiring mode in isolated SAN cells (Fig. 2A). During nonfiring, the cells spontaneously interrupt their electrical activity for up to 1 min and remain at hyperpolarized membrane potentials, followed by recovery to regular action potential firing. The changing between firing and nonfiring occurs at a very slow timescale. Under baseline conditions, nonfiring was less frequently observed in WT cells than in HCN4FEA cells. However, the nonfiring mode could be reliably induced in WT cells by lowering intracellular cAMP concentrations via application of the cholinergic receptor agonist carbachol. Furthermore, the occurrence of nonfiring was drastically increased by incubating the cells with TAT-TRIP8b_nano_, a membrane-permeable peptide that prevents cAMP-dependent activation of HCN channels [63]. Application of the beta-adrenoceptor agonist isoproterenol, which leads to an increase in intracellular cAMP concentrations, completely abolished nonfiring in WT cells. This provides striking evidence for a critical role of HCN4 CDR in regulating the frequency of the nonfiring mode. It leads to the conclusion that cAMP-dependent activation of HCN4 is responsible for terminating nonfiring episodes and maintaining spontaneous action potential firing in SAN pacemaker cells. In contrast, HCN4 CDR is not involved in ANS-induced HR changes, since in the HCN4FEA mutant, the general chronotropic responses to adrenergic and cholinergic stimulation were fully preserved.

The molecular mechanism (or at least a significant part of this mechanism) that causes the switch between firing and nonfiring can be explained by a biophysical phenomenon described as dynamic mode shifts or hysteresis of HCN channels [2, 7, 10, 19, 22, 49, 77, 79]. According to the concept of hysteresis, voltage-dependent activation of HCN4 is a history-dependent process, i.e., the position of the activation curve on the voltage axis (*x*-axis) is determined by long-lasting membrane potentials (Fig. 2B, C). In experimental voltage-clamp settings, such long-lasting membrane potentials can be mimicked by the holding potential [22]. At a relatively depolarized holding potential (− 55 mV), the steady-state activation curve of HCN4 is positioned at extremely hyperpolarized voltages (*V*_0.5_ =  − 100 mV). Conversely, when a relatively hyperpolarized holding potential is applied (− 75 mV), the steady-state activation curve is positioned at extremely depolarized voltages (*V*_0.5_ =  − 78 mV). The described dependence of the steady-state activation of HCN4 on the holding potential has a very interesting consequence for dynamic conditions as observed in the SAN, when the membrane potential slowly drifts. In fact, one would expect that a slowly drifting membrane potential induces a slow and progressive shift of the activation curve within the two maximum limits. This would lead to a left shift of the activation curve towards more negative voltages at increasingly depolarized membrane potentials and to a right shift of the activation curve towards more positive voltages at increasingly hyperpolarized membrane potentials.

Prerequisites for the following model to be true are the slow activation and deactivation kinetics of HCN4 [6]. Until recently, it was completely unclear how voltage-dependent gating of the channel could affect cardiac pacemaking, because the activation and deactivation time constants are 100–1000 times slower than the pacemaker cycle length. Consequently, HCN4 channel activity is barely influenced by the fast voltage changes during individual action potentials (APD90 = 50–80 ms) and is, thus, mainly dependent on the mean membrane potential. Accordingly, the HCN channel–mediated I_f_ current can be considered nearly constant throughout the pacemaker cycle [59], which results in an almost time-independent background current. During SAN action potentials, the flow of the current would therefore depend on the driving force: inwardly directed and depolarizing (as during SDD) or outwardly directed and hyperpolarizing [20].

In the context of the slow changing between firing and nonfiring, however, the purpose of the slow gating kinetics and slow hysteresis becomes clear. The mean membrane potential of SAN pacemaker cells is more depolarized during firing and more hyperpolarized during nonfiring (Fig. 2A). As a consequence of hysteresis, the activation curve of HCN4 slowly shifts towards more negative voltages during firing. Less channels become available, and the HCN4-mediated current becomes smaller which is reflected by progressive hyperpolarization. At a critical point, the shift of the activation curve is completed, and the cells switch into the nonfiring mode. The mean membrane potential abruptly drops to more hyperpolarized values, and, consequently, the activation curve of HCN4 slowly shifts towards more positive voltages. This is accompanied by slow recovery from the hyperpolarized potentials until the threshold for firing is reached. When the cells switch back to the firing mode, the mean membrane potential abruptly jumps to more depolarized values and the cycle repeats. In addition to hysteresis, CDR of HCN4 directly determines the position of the activation curve and, thus, regulates the time point at which the cells switch between the two activity modes. Due to lack of CDR in HCN4FEA cells, the activation curve cannot be shifted to sufficiently positive voltages, which leads to more frequently occurring and longer lasting episodes of nonfiring. The same consideration applies to reduced cAMP levels or acute inhibition of CDR in WT cells following application of carbachol or TAT–TRIP8b_nano_, respectively. In contrast, application of isoproterenol in WT cells leads to a pronounced shift of the activation curve towards more positive voltages, which completely abolishes the switch to the nonfiring mode.

**Fig. 3:**
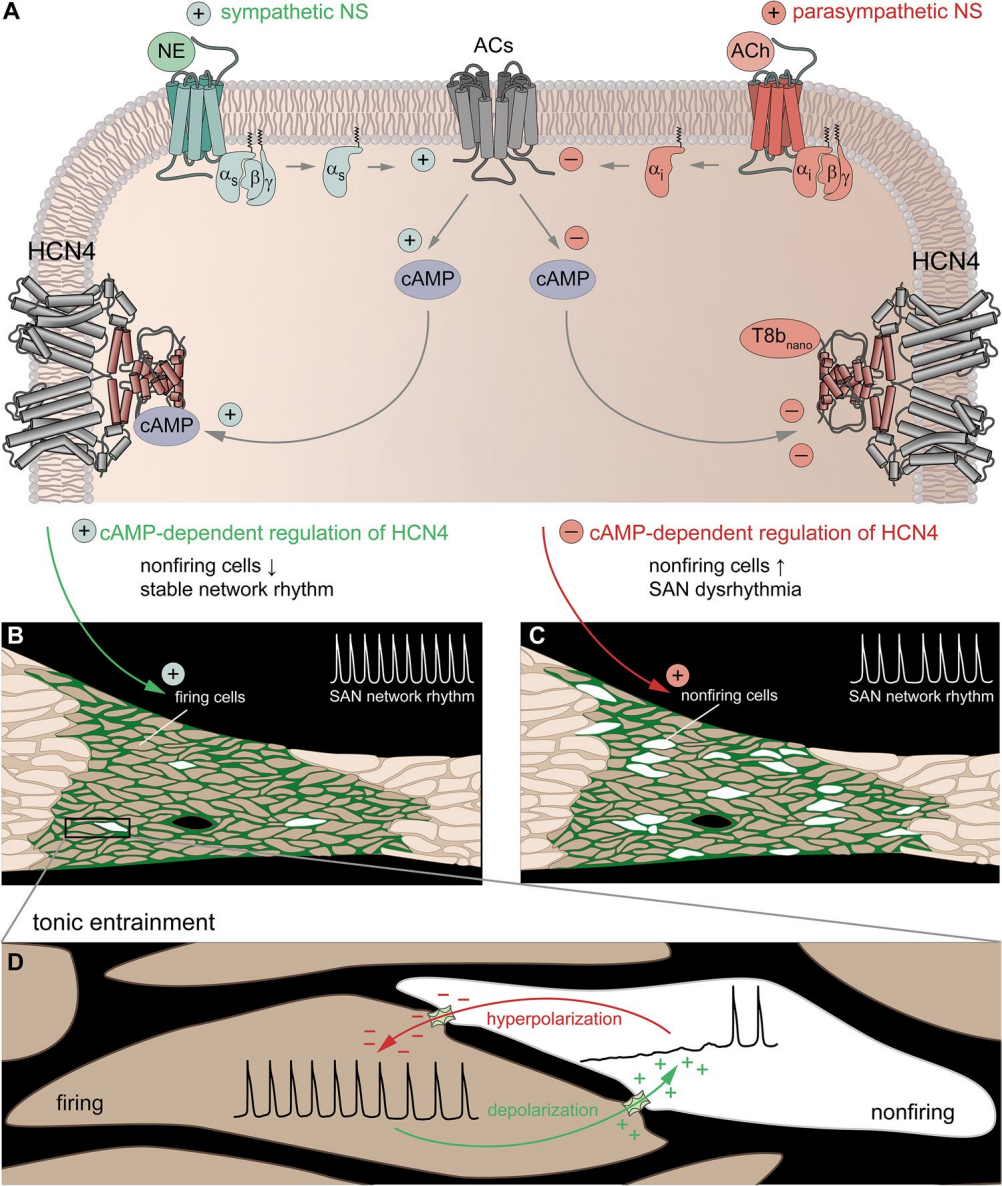
Tonic entrainment in the SAN. (A) Scheme of a SAN cell visualizing the signal transduction pathway following stimulation by the ANS. Gs protein-coupled beta-1-adrenergic receptors (green) are activated by norepinephrine (NE) released from sympathetic nerve terminals. Subsequent Gαs signaling stimulates adenylyl cyclases (ACs, gray) to synthetize cAMP, which directly activates HCN4 channels. Conversely, Gi protein-coupled M2 muscarinic receptors (red) are activated by acetylcholine (ACh) released from vagal nerve terminals. Gαi signaling inhibits ACs and thereby reduces the intracellular cAMP level, leading to a decrease in HCN4 activity. Similar effects are evoked by TRIP8b_nano_, a synthetic peptide that binds to the CNBD of HCN4 and inhibits cAMP-dependent activation of the channel. (B) cAMP-dependent activation of HCN4 reduces the number of nonfiring cells in the SAN, which stabilizes the network rhythm during HR acceleration. (C) Reduction in cAMP-dependent activation of HCN4 increases the number of nonfiring cells in the SAN. Overshooting inhibition leads to bradycardia and SAN dysrhythmia due to destabilization of the network rhythm. (D) The tonic entrainment process takes place between firing cells (left) and neighboring nonfiring cells (right). Pacemaker cells in the nonfiring mode are more hyperpolarized and electrotonically draw the flow of cations from more depolarized neighboring cells in the firing mode via gap junctions. This slightly depolarizes the nonfiring cells (green arrow) and hyperpolarizes the firing cells to the same extent (red arrow)

The actual functional significance of nonfiring pacemaker cells becomes apparent in the intact SAN tissue, where individual pacemaker cells are electrically coupled via gap junctions (Fig. 3). Within this network, intercellular interactions between nonfiring cells and surrounding cells in the firing mode take place, which have an impact on the overall SAN network activity. To answer the question how these long-lasting and “tonic” interactions occur, the following concept was proposed [22]. Pacemaker cells in the nonfiring mode are more hyperpolarized and will electrotonically draw the flow of cations from more depolarized neighboring cells in the firing mode via gap junctions (Fig. 3D). This will slightly depolarize the nonfiring cells and hyperpolarize the firing cells to the same extent. When a new equilibrium is reached, the firing rate of the respective cell cluster will be decreased and, thus, a bradycardic network rhythm emerges. This process is termed tonic entrainment and might be similar to tonic interactions of SAN pacemaker cells and atrial cardiomyocytes at the interface between the SAN and atrial myocardium. Atrial cells have a more hyperpolarized resting membrane potential than SAN pacemaker cells and have been shown to partially suppress the intrinsic pacemaker properties of SAN cells in the border zone [36].

According to this new concept, nonfiring pacemaker cells can be considered a physiologically important, inhibitory component within the SAN network. Their tonic influence on neighboring cells in the firing mode is highly relevant to stabilize SAN network activity and to ensure proper function of the pacemaker process by suppressing overshooting excitation. Under physiological conditions, a stable balance between inhibition (nonfiring cells) and excitation (firing cells) is controlled by CDR of HCN4. This fits well to the concept that inhibitory elements generally increase the stability of electrically active networks [26]. Accordingly, it has been established that inhibitory interneurons are necessary to balance the activity of otherwise unstable neuronal networks in the brain [50, 62]. In the context of nonfiring pacemaker cells, it is likely that such properties of neuronal networks are also attributable to the SAN, where inhibitory control of excitability may be equally essential to ensure a stable function of the pacemaker process. In the absence of HCN4 CDR, however, the precise balance between inhibition and excitation is lost. The resulting overactive inhibition leads to disruption of the pacemaker process and impairment of SAN network function.

## Cardiac phenotype of HCN4FEA mice

**Fig. 4:**
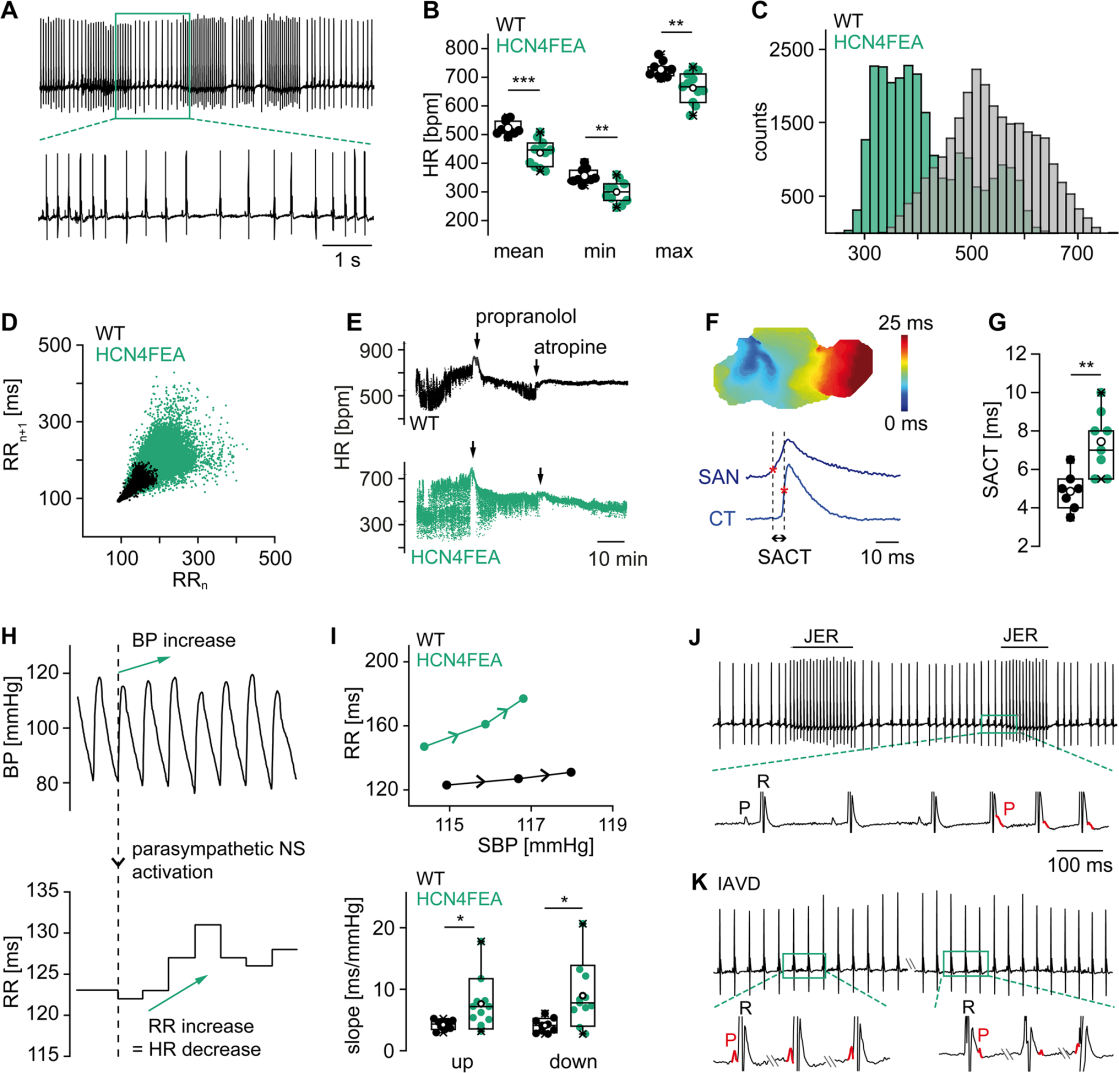
Cardiac phenotype of HCN4FEA mice. (A) Telemetric ECG trace of an HCN4FEA mouse showing severe sinus dysrhythmia. (B) Mean (left), minimum (middle), and maximum (right) heart rate of WT (black) and HCN4FEA mice (green) calculated from 72-h telemetric ECG recordings. (C) Heart rate histograms determined from 72-h recordings. In HCN4FEA mice, the average HR and full HR range is shifted towards lower HR values, demonstrating intrinsic bradycardia. (D) Comet-shaped Poincaré plots display high beat-to-beat dispersion in HCN4FEA mice (green). (E) Tachograms of WT (black) and HCN4FEA mice (green) before and after consecutive injections of propranolol and atropine. (F) Optical imaging measurements of biatrial SAN explants reveal prolonged sinoatrial conduction time (SACT) in HCN4FEA mice. (G) Quantification of SACT determined from optical measurements as shown in panel (F). (H) Combined telemetric blood pressure (upper panel) and ECG recordings (RR intervals, lower panel) used to determine baroreflex sensitivity in vivo. (I) Plot of systolic blood pressure (SBP) and corresponding RR intervals (upper panel) demonstrates a steeper slope of the RR/SBP relationship in HCN4FEA mice (green), reflecting inappropriately enhanced HR responses of the SAN to vagal nerve activity in HCN4FEA mice. Lower panel: quantification of the slope of RR/SBP relations in WT (black) and HCN4FEA mice (green). (J) Telemetric ECG trace of an HCN4FEA mouse during episodes with junctional escape rhythm (JER). (K) Telemetric ECG trace of an HCN4FEA mouse during episodes with isorhythmic AV dissociation (IAVD). Figure is modified from Fenske et al. [22]

At the systemic level, this causes a complex and severe cardiac phenotype of HCN4FEA mice (Fig. 4), which revealed that the lack of HCN4 CDR manifests as intrinsic sinus node dysfunction (Fig. 4A). In vivo telemetric ECG recordings uncovered severe bradycardia, whereas the HR regulation dynamics (HR_max_/HR_min_) and full HR range (HR_max_–HR_min_) were completely preserved (Fig. 4B, C). This further confirms that acceleration and deceleration of HR following ANS activity are mainly driven by ion channels and transporters other than HCN4. However, CDR of HCN4 is crucial for determining absolute HR values and preventing bradycardia. If CDR is missing, too many cells switch into the nonfiring mode and impulse formation and conduction in the SAN are slowed down (Fig. 4F, G). Consequently, the average HR and full HR range shift towards lower HR values (Fig. 4B, C), and intrinsic bradycardia arises. Taken together, CDR of HCN4 plays an important role in setting the intrinsic HR, while it is not required for changing HR per se.

Moreover, severe sinus dysrhythmia was observed in ECG traces of HCN4FEA mice (Fig. 4A and D), which is defined as an irregular HR with large beat-to-beat variations originating in the SAN. Complete autonomic blockade by consecutive injections of atropine and propranolol (Fig. 4E) revealed that a major part of these HR fluctuations was caused by disturbed responsiveness of the SAN to ANS input. This finding was further confirmed by assessing in vivo HR responses to dynamic vagal nerve activity (Fig. 4H, I), which uncovered drastically enhanced HR decreases in HCN4FEA mice, ultimately causing severe bradycardia and sinus pauses. In the absence of CDR, vagal activity inappropriately increases the number of nonfiring pacemaker cells in the SAN, leading to overactive inhibition at the network level. Consequently, HCN4 CDR is required to counteract and dampen the HR-lowering effects of the vagus nerve. This conclusion is in full agreement with the results reported by Kozasa and colleagues [37], and it perfectly fits the well-established role of HCN channels in stabilizing transient changes in the membrane potential [21, 56, 61]. Since a significant proportion of the channels is constitutively open at voltages near the RMP, the presence of HCN channels per se stabilizes the RMP by lowering the membrane resistance [7, 45, 55–57]. Consequently, any given input current causes a smaller change in membrane potential than it would do in the absence of functional HCN channels.

Another key finding from HCN4FEA mice was that overshooting inhibition due to an enhanced proportion of nonfiring cells led to chronotropic incompetence of the SAN, which is defined as the inability to reach the normal maximum firing rate (Fig. 4B, C). In contrast, results from an in vivo electrophysiological study [28] demonstrated that AV-nodal conduction properties were unaffected and the general dromotropic function was normal, indicating that in the absence of HCN4 CDR, the AVN remains chronotropically competent. In-depth analysis of ECG recordings uncovered the presence of two distinct types of arrhythmias, namely isorhythmic AV dissociation (IAVD) and junctional escape rhythm (JER) (Fig. 4J, K). During IAVD, two independent pacemakers are synchronously active, i.e., the SAN activates the atria and, a second, subsidiary pacemaker activates the ventricles independently from SAN function (Fig. 4K). As a result, atrial and ventricular complexes dissociate from each other. However, the atrial and ventricular intervals remain isorhythmic because the firing rates of the SAN and the subsidiary pacemaker are roughly equal. During JER, the firing rate of the subsidiary pacemaker is faster than that of the SAN and, thus, effectively suppresses SAN function. Consequently, the subsidiary pacemaker first activates the ventricles, followed by retrograde activation of the atria (Fig. 4J). In HCN4FEA mice, both arrhythmias were spontaneously induced during HR acceleration by the sympathetic nervous system (SNS). Their occurrence is attributable to the fact that the coordinated activity of the SAN and AVN is disturbed. Upon SNS activation, the firing rate of the SAN cannot be sufficiently increased due to chronotropic incompetence. In contrast, the AVN is chronotropically competent and its firing rate can be increased normally. IAVD occurs when the AVN firing rate is accelerated and approximately matches that of the SAN. JER occurs when the AVN firing rate exceeds that of the SAN and fully suppresses SAN activity.

Taken together, the role of HCN4 CDR in the SAN is to stabilize the electrical activity of the network and to protect the SAN from potentially destabilizing ANS input. This is achieved by regulating the number of nonfiring pacemaker cells in the SAN, which creates a finely tuned balance between inhibitory and excitatory elements. Ultimately, this balance is necessary to create and stabilize the intrinsic responsiveness of the SAN network to ANS activity. Conversely, in the absence of HCN4 CDR, the electrical activity of the SAN becomes instable, and deep bradycardia, severe sinus dysrhythmia, pronounced HR fluctuations and escape arrhythmias occur.

## Nonfiring pacemaker cells in other mouse models

For more than 15 years, various groups have been reporting that sinoatrial node pacemaker cells isolated from different species, including mice, guinea pigs, rabbits, and humans, do not always fire spontaneous action potentials. After long being mistaken as experimental artifacts, it has by now been more frequently suggested that the “silent” phenotype of these cells actually arises from a physiological background. Most of these studies were performed on pacemaker cells isolated from mice. The different states of quiescence could either be observed already under baseline conditions in WT cells, appeared in different knock-out or knock-in models, or were induced or abolished by drug application [1, 4, 12, 13, 22, 32, 35, 38, 43, 51, 67, 68, 70, 73]. A common characteristic of these studies is that beta-adrenergic signalling can stimulate quiescent pacemaker cells to resume to spontaneous firing. It appears, however, that not only CDR of HCN4, but also other redundant mechanisms exist to ensure this transition. Accordingly, a recent study suggested an additional role of L-type Ca_V_1.3 channels in initiating and maintaining automaticity in silent SAN cells upon beta-adrenergic stimulation [43]. Nevertheless, the manifestations of the quiescent states reported in the abovementioned studies were very heterogeneous, especially with respect to the time scale at which they occurred. In isolated SAN cells of HCN4FEA mice and, most importantly, also in those of WT mice, the periodic changing between firing and nonfiring occurred very slowly. Therefore, this unexpected discovery was only possible by means of ultra-stable long-term perforated patch clamp recordings and in the context of the HCN4FEA mutant in which nonfiring is heavily exaggerated [22]. The requirement of such difficult experimental conditions might be a reason why this particular phenomenon has not been clearly characterized and investigated earlier. However, the results of some of the previously published HCN4 mouse models give first insights into the cellular effects of missing or mutated HCN4 channels (Table 1). Isolation of single SAN cells from inducible HCN4 knockout mice uncovered a large fraction of quiescent cells with hyperpolarized membrane potentials that did not show any spontaneous electrical activity [29, 33]. Experiments with SAN cells isolated from cAMP-insensitive hHCN4-573X mice revealed a diverse cellular phenotype with cells being arrhythmic, changing between spontaneous firing and silent states, or fully lacking electrical automaticity [1]. This is in line with the spontaneous alternation between firing and nonfiring in HCN4FEA pacemaker cells [22]. In WT cells, spontaneous and continuous rhythmic action potentials are predominantly observed, which are only very rarely interrupted by short nonfiring episodes. Altogether, these observations suggest that in the complete absence of HCN4 channels, pacemaker cells enter a state of deep hyperpolarization. In this state, the membrane potential is far too negative to reach the threshold for action potential firing, and the cells remain quiescent [29, 33]. In the presence of cAMP-insensitive, but otherwise functional HCN4 channels, the MDP slowly oscillates at the borderline of the firing threshold due to mode shifts of HCN4, and the cells regularly switch between the firing and nonfiring mode [22]. In WT cells with fully intact HCN4 channels, the membrane potential only rarely drops below the threshold for firing, and the cells predominantly remain in the firing mode. In summary, these observations corroborate the newly described role of HCN4 and its cAMP-dependent regulation in controlling the transition between the two activity modes, which has important physiological implications for the general SAN pacemaker process and HR regulation by the ANS.

## Role of voltage-gated calcium and potassium channels in cardiac pacemaking

As discussed above, evidence is increasing that CDR of HCN4 is not the main mechanism in SAN pacemaker cells to mediate the chronotropic effect. This leads to the conclusion that acceleration and deceleration of HR following ANS activity are mainly driven by ion channels and/or transporters other than HCN4. Since several proteins associated with sinoatrial pacemaking are targeted and modulated by ANS activity, there are various potential candidates to possibly mediate the chronotropic effect at the subcellular level. Upon release of norepinephrine (NE) from sympathetic nerve terminals, the stimulating adenylyl cyclase–cAMP–protein kinase A cascade is activated. Increasing activity of protein kinase A (PKA) leads to phosphorylation and activation of various target proteins that have been shown to be involved in pacemaker activity [46]. Among them are L-type Ca^2+^ channels [69] and delayed rectifier potassium channels [40]. Furthermore, it is known that sympathetic activity stimulates Ca^2+^/calmodulin-dependent kinase II (CaMKII) [23, 75, 76], which shares several downstream targets with PKA including L-type Ca^2+^ channels [71].

In line with this, investigation of voltage-gated Ca^2+^ channels has revealed that I_Ca,L_ is enhanced by PKA-dependent [47, 69] and CaMKII-dependent phosphorylation [18, 47, 71]. Accordingly, experiments with Ca_V_1.3 knockout mice demonstrated slower firing rates and a reduced slope of SDD in isolated SAN pacemaker cells [4, 48]. However, augmentation of I_Ca,L_ alone seems not to be sufficient to induce a physiological increase in action potential firing rate following beta-adrenergic stimulation [39, 72]. Furthermore, a potential isoproterenol-induced effect on I_Ca,T_ in the SAN is not yet completely resolved [24, 41]. In addition, a possible role in modulating the firing frequency of rabbit SAN pacemaker cells has been described for the total delayed rectifier potassium current (I_K_) [40]. Application of isoproterenol caused a PKA-dependent increase in I_K_ amplitude and rate of deactivation, together with a negative shift in the activation curve by approximately 10 mV. This was accompanied by a moderate increase in the firing frequency by 16%. Moreover, pharmacological blockade of I_K,r_ by application of E-4031 evoked a significant decrease in the spontaneous pacing rate of Langendorff-perfused, isolated mouse hearts [13].

In summary, there are several redundant processes involved, which have all been shown to be important for HR modulation when studied individually. However, it is well possible that some of these processes represent backup mechanisms with the purpose to maintain proper chronotropic responses if one or several other components should fail. Therefore, a single main subcellular mechanism in SAN pacemaker cells to mediate HR regulation by the ANS has not yet been clearly identified.

## Conclusion

Forty years after the discovery of the I_f_ current, the originally postulated concept that CDR of HCN4 is responsible for mediating the classical chronotropic effect could still not be reliably confirmed. In addition, the partially conflicting results and conclusions derived from studies on different HCN4 mouse models have made it difficult to clearly unravel the physiological purpose of this key ion channel in the SAN pacemaker process. In the past few years, however, it became more and more accepted that CDR of HCN4 is not required for changing HR per se. Instead, it has been shown that HCN4 CDR is responsible for setting the intrinsic HR and for protecting the SAN from potentially harmful ANS input. This is achieved by creating and maintaining a finely tuned balance between inhibition and excitation at the network level, which relies on tightly controlling the number of nonfiring pacemaker cells within the SAN. As a result, the electrical activity of the SAN network is stabilized, which enables normal physiological HR responses to ANS activity. Altogether, these findings provide new evidence that mechanisms other than HCN4 CDR are responsible for mediating the classical chronotropic effect, and they define a novel concept for HCN4 function in the central SAN pacemaker process.

## Data Availability

Data sharing not applicable to this article as no datasets were generated or analyzed during the current study.
